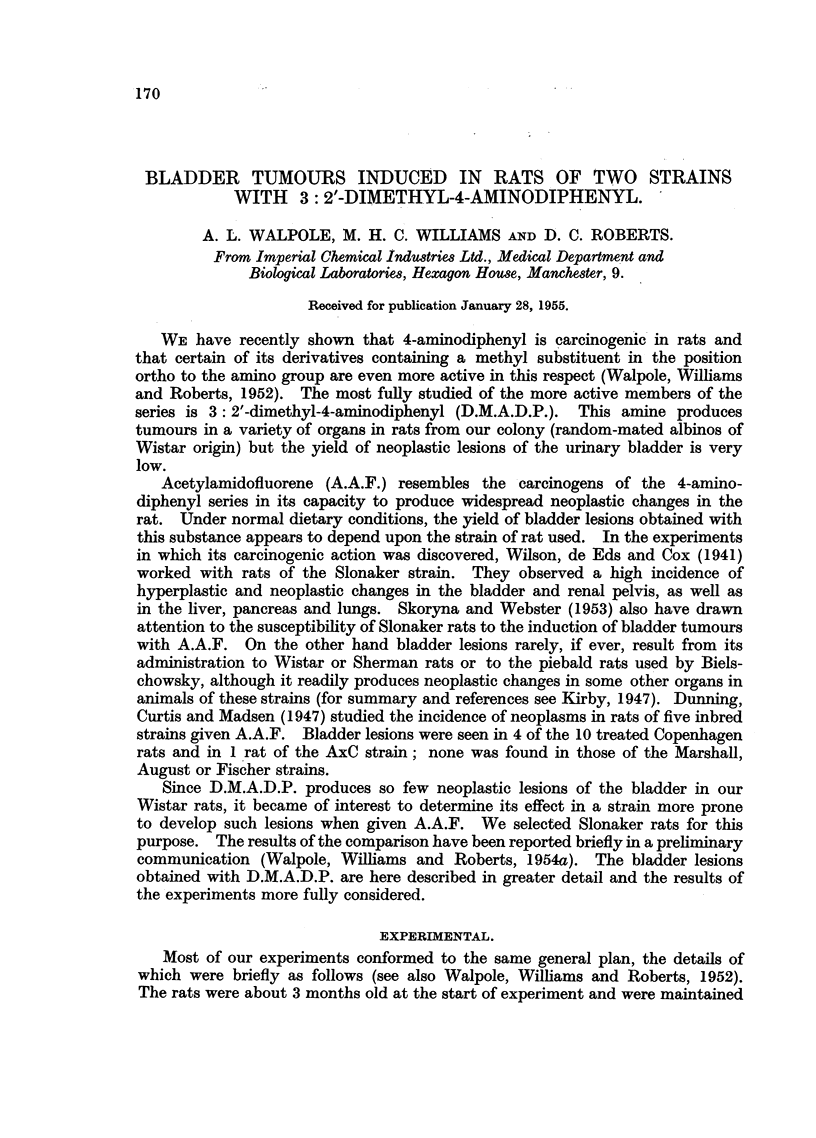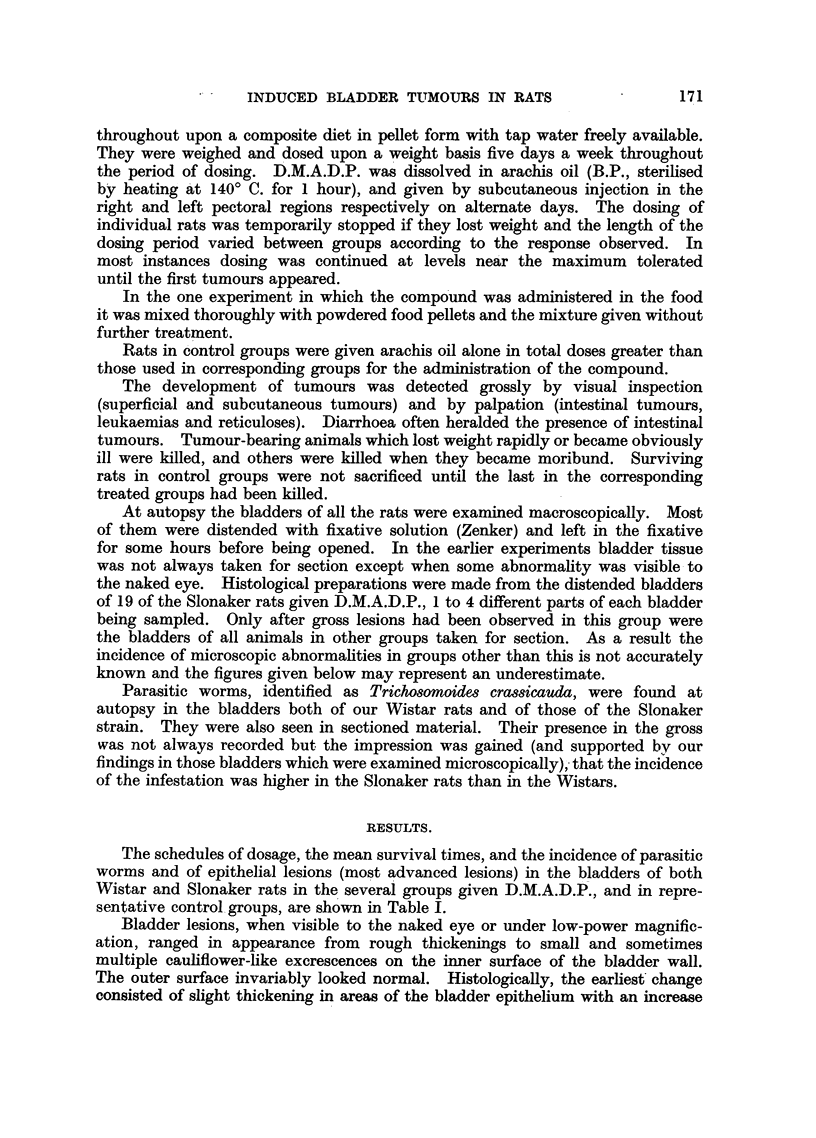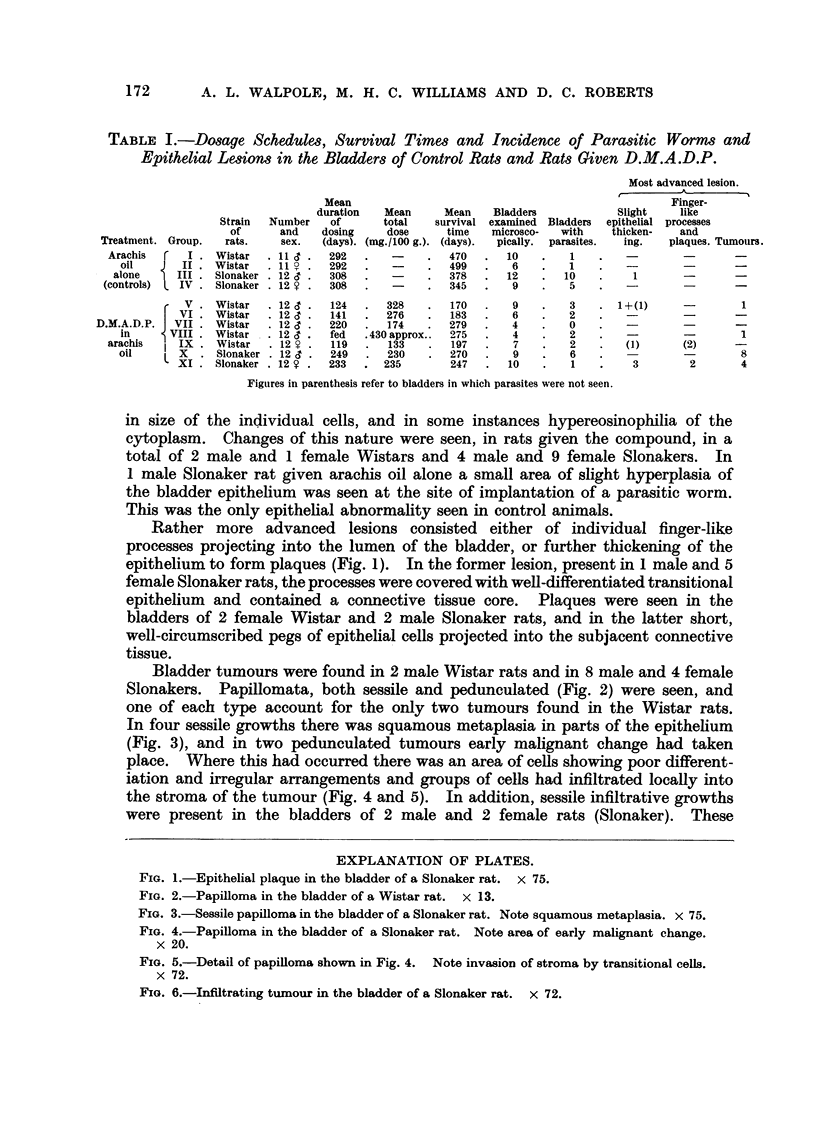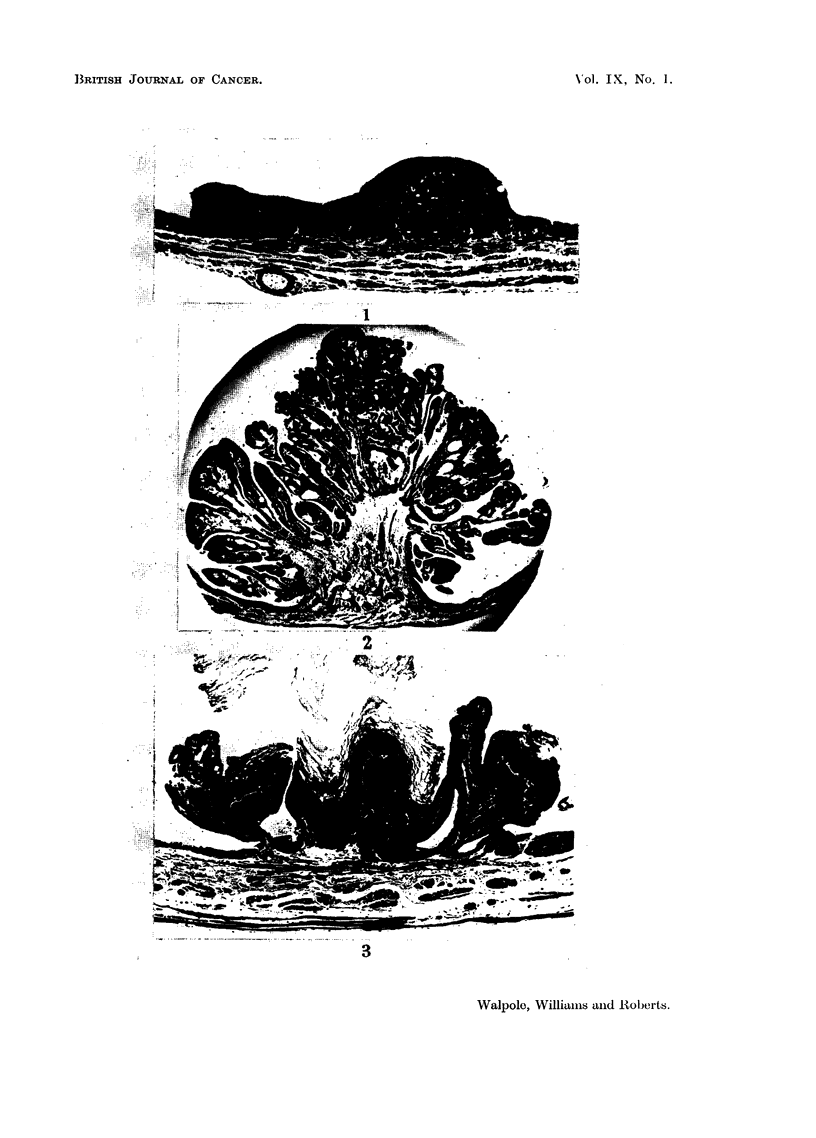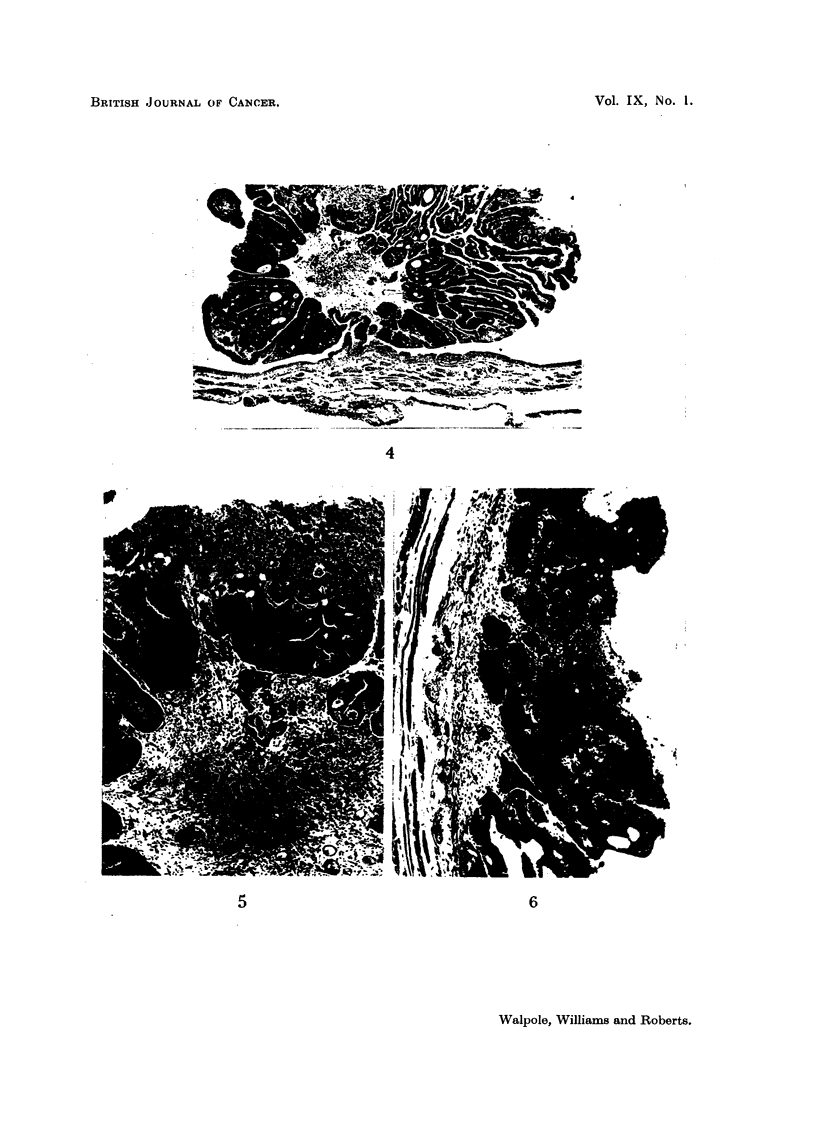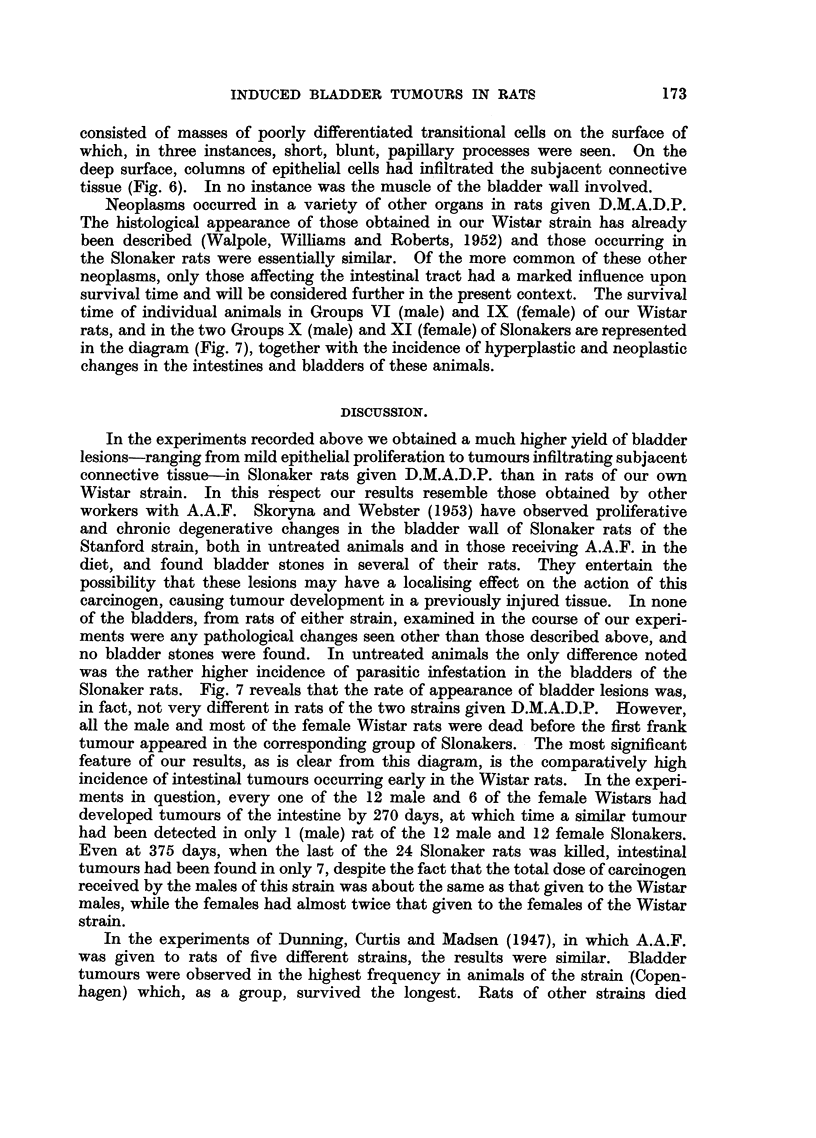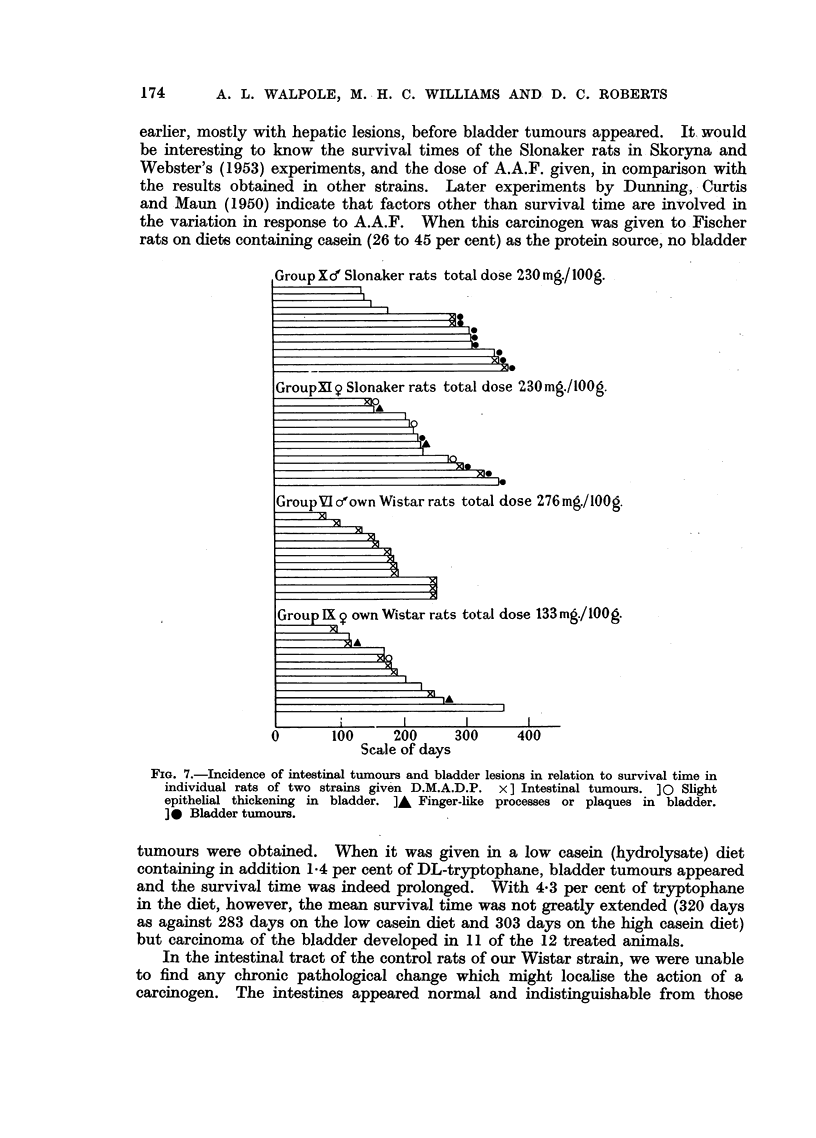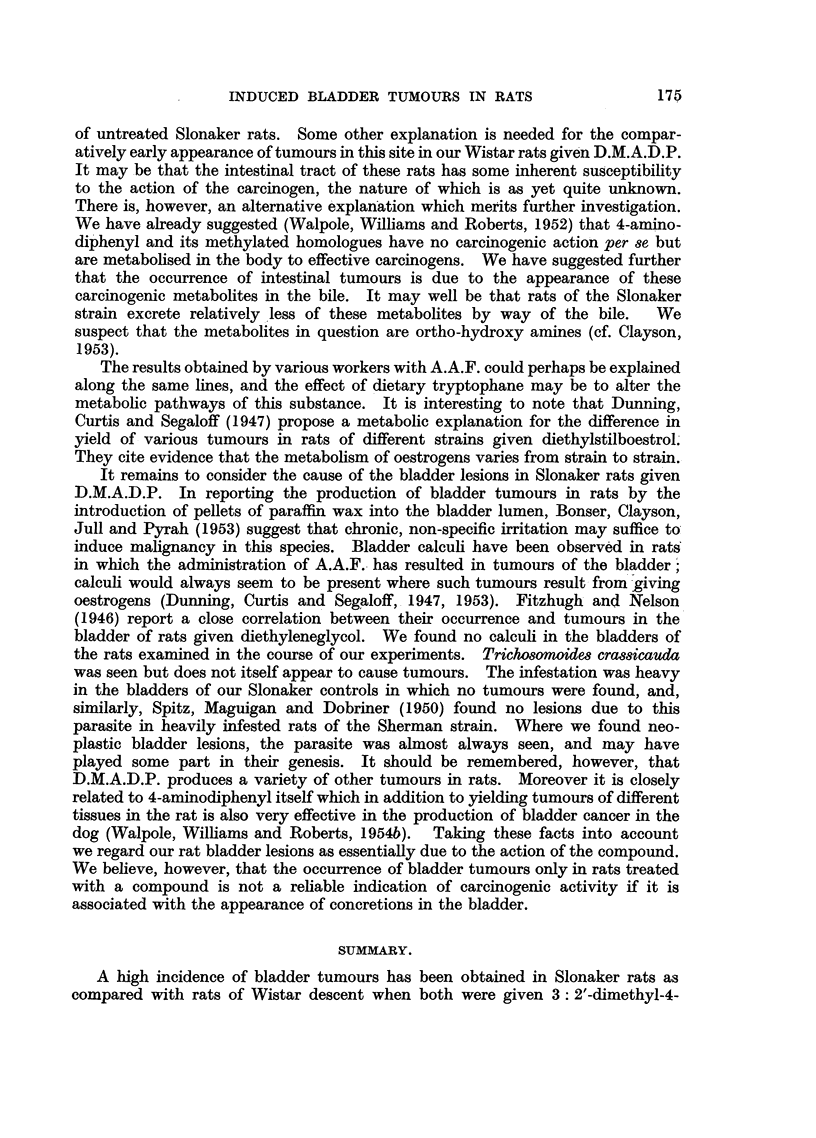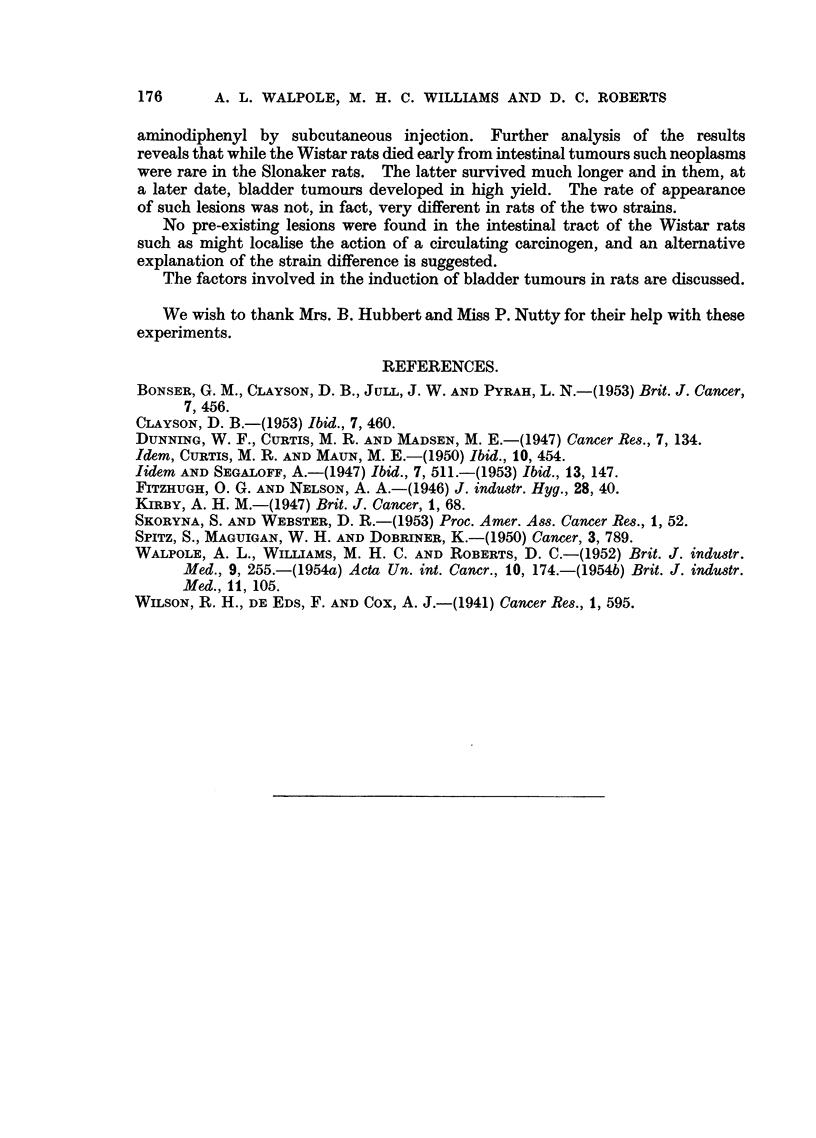# Bladder Tumours Induced in Rats of Two Strains with 3:2′-Dimethyl-4-Aminodiphenyl

**DOI:** 10.1038/bjc.1955.13

**Published:** 1955-03

**Authors:** A. L. Walpole, M. H. C. Williams, D. C. Roberts

## Abstract

**Images:**


### BLADDER TUMOURS INDUCED IN RATS OF TWO STRAINS WITH 3 : 2′-DIMETHYL-4-AMINODIPHENYL.

A. L. WALPOLE, M. H. C. WILLIAMS AND D. C. ROBERTS.

*From Imperial Chemical Industries Ltd., Medical Department and Biological Laboratories, Hexagon House, Manchester, 9.*

Received for publication January 28, 1955.

WE have recently shown that 4-aminodiphenyl is carcinogenic in rats and that certain of its derivatives containing a methyl substituent in the position ortho to the amino group are even more active in this respect (Walpole, Williams and Roberts, 1952). The most fully studied of the more active members of the series is 3 : 2′-dimethyl-4-aminodiphenyl (D.M.A.D.P.). This amine produces tumours in a variety of organs in rats from our colony (random-mated albinos of Wistar origin) but the yield of neoplastic lesions of the urinary bladder is very low.

Acetylamidofluorene (A.A.F.) resembles the carcinogens of the 4-amino-diphenyl series in its capacity to produce widespread neoplastic changes in the rat. Under normal dietary conditions, the yield of bladder lesions obtained with this substance appears to depend upon the strain of rat used. In the experiments in which its carcinogenic action was discovered, Wilson, de Eds and Cox (1941) worked with rats of the Slonaker strain. They observed a high incidence of hyperplastic and neoplastic changes in the bladder and renal pelvis, as well as in the liver, pancreas and lungs. Skoryna and Webster (1953) also have drawn attention to the susceptibility of Slonaker rats to the induction of bladder tumours with A.A.F. On the other hand bladder lesions rarely, if ever, result from its administration to Wistar or Sherman rats or to the piebald rats used by Bielschowsky, although it readily produces neoplastic changes in some other organs in animals of these strains (for summary and references see Kirby, 1947). Dunning, Curtis and Madsen (1947) studied the incidence of neoplasms in rats of five inbred strains given A.A.F. Bladder lesions were seen in 4 of the 10 treated Copenhagen rats and in 1 rat of the AxC strain ; none was found in those of the Marshall, August or Fischer strains.

Since D.M.A.D.P. produces so few neoplastic lesions of the bladder in our Wistar rats, it became of interest to determine its effect in a strain more prone to develop such lesions when given A.A.F. We selected Slonaker rats for this purpose. The results of the comparison have been reported briefly in a preliminary communication (Walpole, Williams and Roberts, 1954*a*). The bladder lesions obtained with D.M.A.D.P. are here described in greater detail and the results of the experiments more fully considered.

### EXPERIMENTAL.

Most of our experiments conformed to the same general plan, the details of which were briefly as follows (see also Walpole, Williams and Roberts, 1952). The rats were about 3 months old at the start of experiment and were maintained throughout upon a composite diet in pellet form with tap water freely available. They were weighed and dosed upon a weight basis five days a week throughout the period of dosing. D.M.A.D.P. was dissolved in arachis oil (B.P., sterilised by heating at 140° C. for 1 hour), and given by subcutaneous injection in the right and left pectoral regions respectively on alternate days. The dosing of individual rats was temporarily stopped if they lost weight and the length of the dosing period varied between groups according to the response observed. In most instances dosing was continued at levels near the maximum tolerated until the first tumours appeared.

In the one experiment in which the compound was administered in the food it was mixed thoroughly with powdered food pellets and the mixture given without further treatment.

Rats in control groups were given arachis oil alone in total doses greater than those used in corresponding groups for the administration of the compound.

The development of tumours was detected grossly by visual inspection (superficial and subcutaneous tumours) and by palpation (intestinal tumours, leukaemias and reticuloses). Diarrhoea often heralded the presence of intestinal tumours. Tumour-bearing animals which lost weight rapidly or became obviously ill were killed, and others were killed when they became moribund. Surviving rats in control groups were not sacrificed until the last in the corresponding treated groups had been killed.

At autopsy the bladders of all the rats were examined macroscopically. Most of them were distended with fixative solution (Zenker) and left in the fixative for some hours before being opened. In the earlier experiments bladder tissue was not always taken for section except when some abnormality was visible to the naked eye. Histological preparations were made from the distended bladders of 19 of the Slonaker rats given D.M.A.D.P., 1 to 4 different parts of each bladder being sampled. Only after gross lesions had been observed in this group were the bladders of all animals in other groups taken for section. As a result the incidence of microscopic abnormalities in groups other than this is not accurately known and the figures given below may represent an underestimate.

Parasitic worms, identified as *Trichosomoides crassicauda,* were found at autopsy in the bladders both of our Wistar rats and of those of the Slonaker strain. They were also seen in sectioned material. Their presence in the gross was not always recorded but the impression was gained (and supported by our findings in those bladders which were examined microscopically), that the incidence of the infestation was higher in the Slonaker rats than in the Wistars.

### RESULTS.

The schedules of dosage, the mean survival times, and the incidence of parasitic worms and of epithelial lesions (most advanced lesions) in the bladders of both Wistar and Slonaker rats in the several groups given D.M.A.D.P., and in representative control groups, are shown in Table I.

Bladder lesions, when visible to the naked eye or under low-power magnification, ranged in appearance from rough thickenings to small and sometimes multiple cauliflower-like excrescences on the inner surface of the bladder wall. The outer surface invariably looked normal. Histologically, the earliest change consisted of slight thickening in areas of the bladder epithelium with an increase

TABLE I.—*Dosage Schedules, Survival Times and Incidence of Parasitic Worms and Epithelial Lesions in the Bladders of Control Rats and Rats Given D.M.A.D.P.*

| Treatment. | Group. | Strain of rats. | Number and sex. | Mean duration of dosing (days). | Mean total dose (mg./100 g.). | Mean survival time (days). | Bladders examined microscopically. | Bladders with parasites. | Most advanced lesion. Slight epithelial thickening. | Most advanced lesion. Finger-like processes and plaques. | Tumours. |
|---|---|---|---|---|---|---|---|---|---|---|---|
| Arachis oil alone (controls) | I | Wistar | 11 ♂ | 292 | — | 470 | 10 | 1 | — | — | — |
| | II | Wistar | 11 ♀ | 292 | — | 499 | 6 | 1 | — | — | — |
| | III | Slonaker | 12 ♂ | 308 | — | 378 | 12 | 10 | 1 | — | — |
| | IV | Slonaker | 12 ♀ | 308 | — | 345 | 9 | 5 | — | — | — |
| D.M.A.D.P. in arachis oil | V | Wistar | 12 ♂ | 124 | 328 | 170 | 9 | 3 | 1+(1) | — | 1 |
| | VI | Wistar | 12 ♂ | 141 | 276 | 183 | 6 | 2 | — | — | — |
| | VII | Wistar | 12 ♂ | 220 | 174 | 279 | 4 | 0 | — | — | — |
| | VIII | Wistar | 12 ♂ | fed | 430 approx. | 275 | 4 | 2 | — | — | 1 |
| | IX | Wistar | 12 ♀ | 119 | 133 | 197 | 7 | 2 | (1) | (2) | — |
| | X | Slonaker | 12 ♂ | 249 | 230 | 270 | 9 | 6 | — | — | 8 |
| | XI | Slonaker | 12 ♀ | 233 | 235 | 247 | 10 | 1 | 3 | 2 | 4 |

Figures in parenthesis refer to bladders in which parasites were not seen.

in size of the individual cells, and in some instances hypereosinophilia of the cytoplasm. Changes of this nature were seen, in rats given the compound, in a total of 2 male and 1 female Wistars and 4 male and 9 female Slonakers. In 1 male Slonaker rat given arachis oil alone a small area of slight hyperplasia of the bladder epithelium was seen at the site of implantation of a parasitic worm. This was the only epithelial abnormality seen in control animals.

Rather more advanced lesions consisted either of individual finger-like processes projecting into the lumen of the bladder, or further thickening of the epithelium to form plaques (Fig. 1). In the former lesion, present in 1 male and 5 female Slonaker rats, the processes were covered with well-differentiated transitional epithelium and contained a connective tissue core. Plaques were seen in the bladders of 2 female Wistar and 2 male Slonaker rats, and in the latter short, well-circumscribed pegs of epithelial cells projected into the subjacent connective tissue.

Bladder tumours were found in 2 male Wistar rats and in 8 male and 4 female Slonakers. Papillomata, both sessile and pedunculated (Fig. 2) were seen, and one of each type account for the only two tumours found in the Wistar rats. In four sessile growths there was squamous metaplasia in parts of the epithelium (Fig. 3), and in two pedunculated tumours early malignant change had taken place. Where this had occurred there was an area of cells showing poor differentiation and irregular arrangements and groups of cells had infiltrated locally into the stroma of the tumour (Fig. 4 and 5). In addition, sessile infiltrative growths were present in the bladders of 2 male and 2 female rats (Slonaker). These

EXPLANATION OF PLATES.

FIG. 1.—Epithelial plaque in the bladder of a Slonaker rat. × 75.

FIG. 2.—Papilloma in the bladder of a Wistar rat. × 13.

FIG. 3.—Sessile papilloma in the bladder of a Slonaker rat. Note squamous metaplasia. × 75.

FIG. 4.—Papilloma in the bladder of a Slonaker rat. Note area of early malignant change. × 20.

FIG. 5.—Detail of papilloma shown in Fig. 4. Note invasion of stroma by transitional cells. × 72.

FIG. 6.—Infiltrating tumour in the bladder of a Slonaker rat. × 72.

1

2

3

Walpole, Williams and Roberts.

4

5

6

Walpole, Williams and Roberts.

consisted of masses of poorly differentiated transitional cells on the surface of which, in three instances, short, blunt, papillary processes were seen. On the deep surface, columns of epithelial cells had infiltrated the subjacent connective tissue (Fig. 6). In no instance was the muscle of the bladder wall involved.

Neoplasms occurred in a variety of other organs in rats given D.M.A.D.P. The histological appearance of those obtained in our Wistar strain has already been described (Walpole, Williams and Roberts, 1952) and those occurring in the Slonaker rats were essentially similar. Of the more common of these other neoplasms, only those affecting the intestinal tract had a marked influence upon survival time and will be considered further in the present context. The survival time of individual animals in Groups VI (male) and IX (female) of our Wistar rats, and in the two Groups X (male) and XI (female) of Slonakers are represented in the diagram (Fig. 7), together with the incidence of hyperplastic and neoplastic changes in the intestines and bladders of these animals.

### DISCUSSION.

In the experiments recorded above we obtained a much higher yield of bladder lesions—ranging from mild epithelial proliferation to tumours infiltrating subjacent connective tissue—in Slonaker rats given D.M.A.D.P. than in rats of our own Wistar strain. In this respect our results resemble those obtained by other workers with A.A.F. Skoryna and Webster (1953) have observed proliferative and chronic degenerative changes in the bladder wall of Slonaker rats of the Stanford strain, both in untreated animals and in those receiving A.A.F. in the diet, and found bladder stones in several of their rats. They entertain the possibility that these lesions may have a localising effect on the action of this carcinogen, causing tumour development in a previously injured tissue. In none of the bladders, from rats of either strain, examined in the course of our experiments were any pathological changes seen other than those described above, and no bladder stones were found. In untreated animals the only difference noted was the rather higher incidence of parasitic infestation in the bladders of the Slonaker rats. Fig. 7 reveals that the rate of appearance of bladder lesions was, in fact, not very different in rats of the two strains given D.M.A.D.P. However, all the male and most of the female Wistar rats were dead before the first frank tumour appeared in the corresponding group of Slonakers. The most significant feature of our results, as is clear from this diagram, is the comparatively high incidence of intestinal tumours occurring early in the Wistar rats. In the experiments in question, every one of the 12 male and 6 of the female Wistars had developed tumours of the intestine by 270 days, at which time a similar tumour had been detected in only 1 (male) rat of the 12 male and 12 female Slonakers. Even at 375 days, when the last of the 24 Slonaker rats was killed, intestinal tumours had been found in only 7, despite the fact that the total dose of carcinogen received by the males of this strain was about the same as that given to the Wistar males, while the females had almost twice that given to the females of the Wistar strain.

In the experiments of Dunning, Curtis and Madsen (1947), in which A.A.F. was given to rats of five different strains, the results were similar. Bladder tumours were observed in the highest frequency in animals of the strain (Copenhagen) which, as a group, survived the longest. Rats of other strains died earlier, mostly with hepatic lesions, before bladder tumours appeared. It would be interesting to know the survival times of the Slonaker rats in Skoryna and Webster's (1953) experiments, and the dose of A.A.F. given, in comparison with the results obtained in other strains. Later experiments by Dunning, Curtis and Maun (1950) indicate that factors other than survival time are involved in the variation in response to A.A.F. When this carcinogen was given to Fischer rats on diets containing casein (26 to 45 per cent) as the protein source, no bladder

FIG. 7.—Incidence of intestinal tumours and bladder lesions in relation to survival time in individual rats of two strains given D.M.A.D.P. ×] Intestinal tumours. ]○ Slight epithelial thickening in bladder. ]▲ Finger-like processes or plaques in bladder. ]● Bladder tumours.

tumours were obtained. When it was given in a low casein (hydrolysate) diet containing in addition 1·4 per cent of DL-tryptophane, bladder tumours appeared and the survival time was indeed prolonged. With 4·3 per cent of tryptophane in the diet, however, the mean survival time was not greatly extended (320 days as against 283 days on the low casein diet and 303 days on the high casein diet) but carcinoma of the bladder developed in 11 of the 12 treated animals.

In the intestinal tract of the control rats of our Wistar strain, we were unable to find any chronic pathological change which might localise the action of a carcinogen. The intestines appeared normal and indistinguishable from those of untreated Slonaker rats. Some other explanation is needed for the comparatively early appearance of tumours in this site in our Wistar rats given D.M.A.D.P. It may be that the intestinal tract of these rats has some inherent susceptibility to the action of the carcinogen, the nature of which is as yet quite unknown. There is, however, an alternative explanation which merits further investigation. We have already suggested (Walpole, Williams and Roberts, 1952) that 4-aminodiphenyl and its methylated homologues have no carcinogenic action *per se* but are metabolised in the body to effective carcinogens. We have suggested further that the occurrence of intestinal tumours is due to the appearance of these carcinogenic metabolites in the bile. It may well be that rats of the Slonaker strain excrete relatively less of these metabolites by way of the bile. We suspect that the metabolites in question are ortho-hydroxy amines (cf. Clayson, 1953).

The results obtained by various workers with A.A.F. could perhaps be explained along the same lines, and the effect of dietary tryptophane may be to alter the metabolic pathways of this substance. It is interesting to note that Dunning, Curtis and Segaloff (1947) propose a metabolic explanation for the difference in yield of various tumours in rats of different strains given diethylstilboestrol. They cite evidence that the metabolism of oestrogens varies from strain to strain.

It remains to consider the cause of the bladder lesions in Slonaker rats given D.M.A.D.P. In reporting the production of bladder tumours in rats by the introduction of pellets of paraffin wax into the bladder lumen, Bonser, Clayson, Jull and Pyrah (1953) suggest that chronic, non-specific irritation may suffice to induce malignancy in this species. Bladder calculi have been observed in rats in which the administration of A.A.F. has resulted in tumours of the bladder; calculi would always seem to be present where such tumours result from giving oestrogens (Dunning, Curtis and Segaloff, 1947, 1953). Fitzhugh and Nelson (1946) report a close correlation between their occurrence and tumours in the bladder of rats given diethyleneglycol. We found no calculi in the bladders of the rats examined in the course of our experiments. *Trichosomoides crassicauda* was seen but does not itself appear to cause tumours. The infestation was heavy in the bladders of our Slonaker controls in which no tumours were found, and, similarly, Spitz, Maguigan and Dobriner (1950) found no lesions due to this parasite in heavily infested rats of the Sherman strain. Where we found neoplastic bladder lesions, the parasite was almost always seen, and may have played some part in their genesis. It should be remembered, however, that D.M.A.D.P. produces a variety of other tumours in rats. Moreover it is closely related to 4-aminodiphenyl itself which in addition to yielding tumours of different tissues in the rat is also very effective in the production of bladder cancer in the dog (Walpole, Williams and Roberts, 1954*b*). Taking these facts into account we regard our rat bladder lesions as essentially due to the action of the compound. We believe, however, that the occurrence of bladder tumours only in rats treated with a compound is not a reliable indication of carcinogenic activity if it is associated with the appearance of concretions in the bladder.

### SUMMARY.

A high incidence of bladder tumours has been obtained in Slonaker rats as compared with rats of Wistar descent when both were given 3 : 2′-dimethyl-4-

aminodiphenyl by subcutaneous injection. Further analysis of the results reveals that while the Wistar rats died early from intestinal tumours such neoplasms were rare in the Slonaker rats. The latter survived much longer and in them, at a later date, bladder tumours developed in high yield. The rate of appearance of such lesions was not, in fact, very different in rats of the two strains.

No pre-existing lesions were found in the intestinal tract of the Wistar rats such as might localise the action of a circulating carcinogen, and an alternative explanation of the strain difference is suggested.

The factors involved in the induction of bladder tumours in rats are discussed.

We wish to thank Mrs. B. Hubbert and Miss P. Nutty for their help with these experiments.

### REFERENCES.

BONSER, G. M., CLAYSON, D. B., JULL, J. W. AND PYRAH, L. N.—(1953) *Brit. J. Cancer*, **7**, 456.

CLAYSON, D. B.—(1953) *Ibid.*, **7**, 460.

DUNNING, W. F., CURTIS, M. R. AND MADSEN, M. E.—(1947) *Cancer Res.*, **7**, 134.

*Idem*, CURTIS, M. R. AND MAUN, M. E.—(1950) *Ibid.*, **10**, 454.

*Iidem* AND SEGALOFF, A.—(1947) *Ibid.*, **7**, 511.—(1953) *Ibid.*, **13**, 147.

FITZHUGH, O. G. AND NELSON, A. A.—(1946) *J. industr. Hyg.*, **28**, 40.

KIRBY, A. H. M.—(1947) *Brit. J. Cancer*, **1**, 68.

SKORYNA, S. AND WEBSTER, D. R.—(1953) *Proc. Amer. Ass. Cancer Res.*, **1**, 52.

SPITZ, S., MAGUIGAN, W. H. AND DOBRINER, K.—(1950) *Cancer*, **3**, 789.

WALPOLE, A. L., WILLIAMS, M. H. C. AND ROBERTS, D. C.—(1952) *Brit. J. industr. Med.*, **9**, 255.—(1954*a*) *Acta Un. int. Cancr.*, **10**, 174.—(1954*b*) *Brit. J. industr. Med.*, **11**, 105.

WILSON, R. H., DE EDS, F. AND COX, A. J.—(1941) *Cancer Res.*, **1**, 595.